# Long Non-Coding RNAs in Multiple Myeloma

**DOI:** 10.3390/ncrna5010013

**Published:** 2019-01-24

**Authors:** Romana Butova, Petra Vychytilova-Faltejskova, Adela Souckova, Sabina Sevcikova, Roman Hajek

**Affiliations:** 1 Babak Myeloma Group, Department of Pathological Physiology, Faculty of Medicine, Masaryk University, 62500 Brno, Czech Republic; romanabutova@gmail.com (R.B.); 422924@mail.muni.cz (A.S.); 2 Department of Comprehensive Cancer Care, Masaryk Memorial Cancer Institute, 60200 Brno, Czech Republic; vychytilova.petra@seznam.cz; 3 Department of Hematooncology, University Hospital Ostrava and Faculty of Medicine, University Ostrava, 70852 Ostrava, Czech Republic; roman.hajek@fno.cz

**Keywords:** multiple myeloma, long non-coding RNAs, biomarker

## Abstract

Multiple myeloma (MM) is the second most common hematooncological disease of malignant plasma cells in the bone marrow. While new treatment brought unprecedented increase of survival of patients, MM pathogenesis is yet to be clarified. Increasing evidence of expression of long non-coding RNA molecules (lncRNA) linked to development and progression of many tumors suggested their important role in tumorigenesis. To date, over 15,000 lncRNA molecules characterized by diversity of function and specificity of cell distribution were identified in the human genome. Due to their involvement in proliferation, apoptosis, metabolism, and differentiation, they have a key role in the biological processes and pathogenesis of many diseases, including MM. This review summarizes current knowledge of non-coding RNAs (ncRNA), especially lncRNAs, and their role in MM pathogenesis. Undeniable involvement of lncRNAs in MM development suggests their potential as biomarkers.

## 1. Introduction

Multiple myeloma (MM) is the second most common hematological malignancy of plasma cells (PCs), which produce monoclonal immunoglobulin. These cells expand in the bone marrow (BM) replacing normal hematopoiesis. MM is preceded by its premalignant stage, monoclonal gammopathy of undetermined significance (MGUS). Genetic and epigenetic changes contribute to development of a new, aggressive clone of PCs in the BM; malignant PC accumulation can lead to the development of specific symptoms, including hypercalcemia, renal insufficiency, anemia, and osteolytic bone disease (CRAB symptoms). Multiple myeloma is a clinically and biologically heterogeneous disease with survival ranging from a few months to more than 10 years [1]. One of the most important factors related to clinical aggressiveness is the presence of various cytogenetic abnormalities, including translocations and genomic imbalances [2]. Understanding of MM biology evolved rapidly with the introduction of molecular genetics and technological advancements. Chromosomal and molecular abnormalities underlying MM pathogenesis were identified, forming the basis for future targeted therapies.

Long non-coding RNAs (lncRNAs) were considered to be evolutionary accumulated genetic waste until they were proven to be gene expression regulators by modern, highly sensitive genomics platforms. Due to diverse modifications at the levels of transcription, post-transcription, and chromatin remodeling, deregulated expression of lncRNAs seems to reflect origin and progression of many diseases [3]. However, exact mechanisms of lncRNA function are not known. In this paper, we review available information about lncRNAs, and their biogenesis, function, and involvement in MM pathogenesis.

## 2. Definition of Long Non-Coding RNAs

For a long time, the conventional view of gene regulation focused on the central dogma of molecular biology. However, introduction of high-throughput platforms into clinical research revealed the key potential of genes with non-coding features. The human genome sequencing project detected protein-coding genes with 2% whole-genome representation; however, up to 90% of genes are transcriptionally active [4]. These non-coding RNA (ncRNA) molecules are becoming increasingly important, as their roles in various biological processes are being uncovered. Non-coding RNA molecules may be divided into two groups, long non-coding and short non-coding RNAs (sncRNAs), with an arbitrary length of 200 nucleotides (nt) as the division point [5].

Long ncRNAs are a class of non-coding RNAs longer than 200 nt that regulate gene expression [6]. Gene identification and variations in the human genome sequence were presented in the GENCODE project, which analyzed 9277 manually annotated genes producing 14,880 transcripts. Long ncRNAs represent the largest and most variable group of ncRNAs, but their function remains unclear. Signal pathways generating lncRNAs are similar to protein-coding genes pathways, including histone modification profiles, alternative splicing, intron and exon lengths, and 5′-capping. Long ncRNAs are mainly localized in the nucleus [7], their fractions are preferentially processed into small RNAs, and they show higher ratio of degradation. Instability of lncRNAs was expected because of the low lncRNA expression level and the presence of unstable transcripts, such as promoter upstream transcripts (PROMPT) [8]. However, according to recent studies, only 29% of lncRNAs may be considered unstable with a two-hour half-life; 6% of lncRNAs show high stability with a half-life of over 12 h. Classification of these molecules is difficult as many transcripts show a combination of different properties [9].

The GENCODE project output represents an initial categorization of lncRNAs based on their position in DNA in relation to protein-coding genes. It includes antisense RNAs (asRNA), long intergenic RNAs (lincRNAs) localized between two genes, sense overlapping transcripts, sense intronic transcripts derived from a second transcript intron, and processed transcripts without an open reading frame (ORF) [10]. Long ncRNAs can be further divided based on their mode of action into cis- and trans-acting or according to the diverse spectrum of regulatory mechanisms [11]. Finally, lncRNAs associated with telomeres and pseudogenes, which are commonly called nonfunctional copies of genes with disrupted transcription or translation, were previously described [12].

## 3. Comparison to Protein-Coding Genes

The gene structure of long non-coding transcripts is very similar to transcripts of protein-coding genes [10]. Their size ranges from 200 nt to over 100 kb [13]. In most cases, the overall length of lncRNA genes is shorter than the length of protein-coding genes. However, the length of exon and intron regions is longer in lncRNAs, as there is a tendency to have two exons only [10,14]. In addition, lncRNAs have a lower level of sequence conservation than protein-coding messenger RNAs (mRNAs) [15], and their expression levels are roughly 10 times lower than those of mRNAs [9]. Genes for lncRNAs are transcribed by RNA polymerase II/III (Pol II/III), and they undergo post-transcription modifications, just like protein-coding genes [16]. The transcription activity of lncRNAs was proven by triple lysine methylation at position 4 and 36 on histone H3, as it is known that the K4–K36 domain indicates the transcription activity of protein-coding genes [17]. Unlike mRNAs, which are transported from the nucleus to the cytoplasm for initiation of translation, lncRNAs are preferentially localized in the nucleus and usually exhibit high cell- and tissue-specific expression patterns. Moreover, their regulation function is related to localization as they can directly influence gene expression [18].

## 4. Biogenesis of Long Non-Coding RNAs

Long ncRNAs show cell-specific expression and partially positive correlation with the expression of antisense-coding genes. According to the lncRNome database, there are approximately 17,000 human evolutionally conserved lncRNAs [19]. Within the eukaryotic genome, some lncRNA subclasses are transcribed on different places of DNA, including promotors, enhancers, and intergenic regions; others are derived from long primary transcripts in a non-canonical way while generating new lncRNA types. Long ncRNAs exist in a form of independent transcriptional units, as their fractions may play a regulation role as untranslated regions (UTR) of nearby protein-coding genes. The genomic structure of lncRNAs is characterized by the presence of two-exon transcripts; their conservation is a function indicator. Even though most lncRNAs (81%) are weakly conserved at the level of DNA, there are some lncRNAs, which are ultra-conserved. Moreover, 3% of lncRNA families most likely originated more than 300 million years ago. Controversially, lncRNA promotors have more conserved sequences than promotors of protein-coding genes [20].

Most eukaryotic promoters are bidirectional; initiating Pol II can generate a transcript in the sense or antisense direction. Thus, divergent transcripts account for a large proportion of observed lncRNAs. To reach their mature form, nascent RNA transcripts undergo various co-transcriptional and post-transcriptional modifications, such as 5′-capping, polyadenylation, splicing, or chemical base modification [21]. In contrast, recent studies revealed that a number of lncRNAs lack poly(A) tails and are processed in alternative ways. Firstly, the RNase P complex was found to be involved in the 3′-end maturation of some lncRNAs, including *MALAT1* and *NEAT1*. These lncRNAs share two similar structural elements at their 3′ termini, a transfer RNA (tRNA)-like cloverleaf and A/U-rich tracts that form a highly stable triple helix. The RNase P cleaves the tRNA-like structure resulting in a mature lncRNA transcript [22]. While the majority of lncRNAs are transcribed from intergenic regions, some of them were described to be derived from excised introns and lack both 5′ cap and 3′ poly(A) tails [23]. In 2012, many excised introns longer than 200 nt that could accumulate were identified. These introns contain two small nucleolar RNAs (snoRNAs) and are processed from their ends by the small nucleolar ribonucleoprotein (snoRNP) machinery after splicing; internal intronic sequences between snoRNAs are not removed, leading to the formation of lncRNAs with snoRNA ends (sno-lncRNAs) [24]. Circular RNAs (circRNAs) represent a class of lncRNAs that have special 3′- and 5′-end processing. They can be formed through non-sequential splicing of introns or after canonical intron splicing [25,26]. Other lncRNAs with unspliced introns are called exon–intron circRNAs (EIciRNAs). They can promote the transcription of their parental genes through interaction with U1 snRNP and Pol II [27]. Finally, some lncRNAs containing microRNAs (miRNAs) are cleaved by a microprocessor complex to terminate transcription in a polyadenylation-independent manner. These lnc-pri-miRNAs are subsequently processed into miRNAs and unstable, non-polyadenylated lncRNAs [28].

## 5. Localization of Long Non-Coding RNAs

Subcellular patterns of lncRNAs provide fundamental insights into their biology. Long ncRNAs must localize to their site of action; thus, their location in the cell is important. Furthermore, localization into particular areas in the nucleus would suggest different functions—a lncRNA in the nucleus near its site of transcription would suggest that it regulates transcription of a proximal gene [29]. The RNA fluorescent in situ hybridization (FISH) method allows for direct observation of lncRNA localization in the cell. For example, lncRNA *XIST* plays an essential role in the inactivation of the X chromosome. During female development, *XIST* is expressed from the inactive X chromosome and actually coats it [30]. Other examples are *NEAT1*, *MALAT1*, and *MIAT*, which are localized to nuclear bodies [31,32,33], and *GAS5* which shuttles between the nucleus and cytoplasm [34]. The single-molecule RNA FISH technique analyzes the absolute level and subcellular localization of low-abundance lncRNAs; for example, lncRNA *HOXA1* represses the homeobox A1 (*HOXA1*) gene in cis in a subpopulation of cells [35]. A study by Cabili et al. (2015) used single-molecule RNA FISH in a single cell to characterize subcellular localization patterns and the abundance of 34 lncRNAs in three human cell types. This study supported the observation that lncRNAs localize in combination of a set of archetypal localizations including a pattern of predominantly nuclear patterns, which suggests a possibility that these localizations correspond to functional categories. This study showed that most lncRNAs are expressed in a uniform manner from cell to cell and do not remain attached to chromosomes during mitosis [29]. There seems to be a strong nuclear bias toward the localization of lncRNAs (95% of them have a higher nuclear fraction than mRNA). For example, *MEG3* seems to have a similar pattern of localization as *MALAT1* and also seems to co-localize with this molecule, suggesting a functional relationship between these two molecules that were both previously described in various tumors individually [36,37].

Another study used RNA sequencing datasets to create “lncATLAS”, a comprehensive resource of lncRNA localization in human cells. Altogether, 6768 GENCODE-annotated lncRNAs are represented across various compartments of 15 cell lines [38].

## 6. Function of Long Non-Coding RNAs

**Figure 1:**
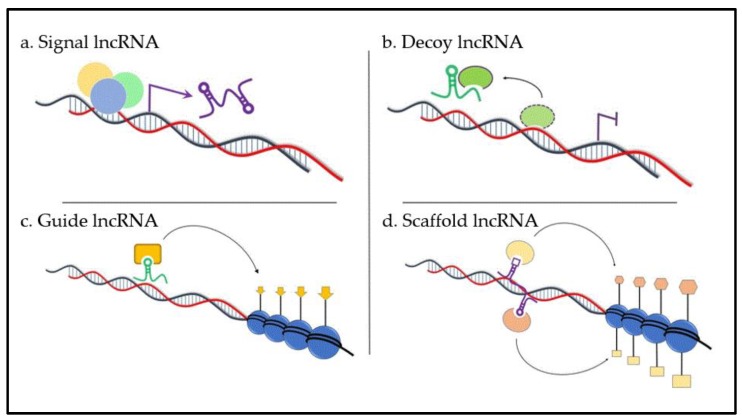
Four archetypes of long non-coding RNA (lncRNA) molecular mechanisms.

Despite new studies of lncRNAs, it is still not known whether all existing lncRNAs have a function. However, it is probable that the majority of lncRNAs are functionally relevant, although heterogeneous in their mode of action. Commonly, the diverse functions of lncRNAs can be divided into four archetypes of molecular mechanisms (Figure 1). Nevertheless, one lncRNA may fulfill several archetypes [39].

Firstly, lncRNAs can serve as molecular signals (archetype I, Figure 1a) as their transcription occurs at a very specific time and place to respond to diverse stimuli. Some of these lncRNAs possess regulatory functions, while others are by-products of transcription or can be associated with chromatin. Recent papers indicate that lncRNAs such as *AIR*, *KCNQ1OT1*, or *XIST* mediate transcriptional silencing of multiple genes by interacting with chromatin and recruiting chromatin modifying machinery [40]. Long ncRNA *H19* is involved in allelic imprinting. It is highly expressed from the *IGF2* locus of the maternal allele during the blastocyst stage and in mesodermal and endodermal tissues, but only in skeletal tissues in adults [41]. Interestingly, *H19* is also a precursor for miR-675 that regulates placental growth [42]. Long ncRNAs are associated with specification of the anterior–posterior body axis and determination of the positional identity of individual cells. While *HOTAIR* is expressed in cells with distal and posterior positional identities, *FRIGIDAIR* has an anterior pattern of expression, and *HOTTIP* is expressed in distal cells [43]. Long ncRNAs also modulate gene activity in response to external stimuli. In the case of DNA damage, p53 can directly induce the expression of lncRNAs *PANDA* and *LINC-p21* leading to cell-cycle arrest [44,45]. Loewer et al. [46] showed that lincRNAs are highly expressed during reprogramming of somatic cells to induced pluripotent stem cells. *LINC-RoR* was proven to be directly targeted by key pluripotency factors SOX2, OCT4, and Nanog.

Secondly, lncRNAs are decoys (archetype II, Figure 1b). These lncRNAs are transcribed and then bind and titrate away protein targets, including transcription factors, chromatin modifiers, and other regulatory factors. They can function in nuclear subdomains or in the cytoplasm. The molecular mechanism of a decoy lncRNA can be represented by telomeric repeat-containing RNA (*TERRA*), which is a product of telomerase transcription. Telomeres are DNA–protein complexes localized at the end of eukaryotic chromosomes, an essence for chromosome stability. *TERRA* shapes an integral part of telomeric heterochromatin as it physically interacts with telomerase through a repetitive sequence complementary to the template sequence of RNA telomerase [47]. Another example of a decoy lncRNA is *PANDA*. It is very sensitive to DNA damage, and its expression is induced ahead of cyclin-dependent kinase inhibitor 1A (CDKN1A). This lncRNA inhibits expression of nuclear transcription factor Y subunit alpha (NF-YA), which activates apoptotic program upon DNA damage [44]. Recently, *GAS5* was identified as a competitor for binding to the DNA-binding domain of the glucocorticoid receptor, thus modulating steroid hormone activity in target tissues [34]. One of the most abundant nuclear lncRNAs in mammalian cells is *MALAT1*. Depletion of this lncRNA alters the localization and activity of splicing factors, and leads to altered patterns of alternative splicing for a set of pre-mRNAs [48]. Interestingly, there is now evidence that mRNA expression can affect the distribution of miRNAs; close relationships between several cancer-related genes and their pseudogenes exist. Specifically, the 3′ untranslated region (UTR) of *PTENP1* RNA was found to bind the same set of regulatory miRNA sequences that target the tumor-suppressor phosphatase and tensin homolog (PTEN) [49]. LncRNA *NRON*, a critical regulator of dephosphorylation of nuclear factor of activated T-cells (NFAT), has a cytoplasmic role and acts as a decoy as well. Inhibition of this lncRNA leads to inactivation of nuclear factor of activated T-cells (NFAT), resulting in migration, tube formation, and proliferation of endothelial cells [50].

Thirdly, lncRNAs are guides (archetype III, Figure 1c). These lncRNAs direct ribonucleoprotein complexes to target genes in cis (close to the place of lncRNA production) or *trans* (in case the genes are a sufficient distance away). Components of this regulation include repressive (polycomb) or activating complexes, e.g., mixed lineage leukemia (MLL) complex, and transcription factors (TFIIβ). Multiple lncRNAs expressed in various cell types bind polycomb repressive complex 2 (PRC2); small interfering RNA (siRNA)-mediated depletion of a number of these molecules led to enrichment of genes normally repressed by PRC2 [51]. LncRNA *AIR* silences the transcription of its target genes via a specific interaction between ncRNA and chromatin at its promoter. Accumulated *AIR* recruits G9a that leads to targeted H3K9 methylation and allelic silencing [52]. Similarly, spreading of *Xist* is accompanied by the recruitment of PRC2 and matrix protein heterogeneous nuclear ribonucleoprotein (hnRNP) U to the inactive X chromosome [53]. In contrast, lncRNA *HOXB-LINC* recruits the SET1/MLL complex and, thus, activates the transcription of *HOXB* gene [54]. In addition, lncRNAs can be involved in the regulation of gene expression by transcriptional co-activator and co-repressor complexes, such as p300 histone acetyltransferase or cyclic AMP response element binding protein (CREB) [55]. In bladder cancer, *UCA1* affects cell-cycle progression through p300 and its co-activator CREB binding via a phosphatidylinositol-3-kinase (PI3K)/ protein kinase B (AKT)-dependent pathway [56].

Fourthly, lncRNAs act as a scaffold (archetype IV, Figure 1d). This is probably the most complex class of lncRNAs; scaffold lncRNAs possess different domains that bind various effector molecules at the same time, thus bringing the transcriptional activators or repressors together in both time and space. As mentioned above, *HOTAIR* binds PRC2, which methylates H3 on lysine 27 to promote gene repression [43]. In addition, the 3′ end was found to interact with the lysine-specific demethylase 1 (LSD1)/CoREST/repressor element 1 silencing transcription factor (REST) complex that demethylates H3 on lysine 4. These observations show that *HOTAIR* can function as a scaffold and bring together different molecules suppressing gene expression [7]. Similarly, *KCNQ1OT1* may perform an analogous function for PRC2 and G9a, mediating H3K27 and H3K9 trimethylation [57], while *ANRIL* secures the interaction between both PRC1 and PRC2 complexes [36,58].

Moreover, certain aspects of lncRNA function can be influenced by the structure of lncRNAs. Even though the structural features are not fully known yet, a study by Smola et al. identified numerous protein interactions based on the structure–function relationship in lncRNAs, as structured RNA motifs determine potential binding sites forming stable interactions, for example, via the appearance of structural elements at the 3′ end of *Xist*. Indeed, this study focused on lncRNA *Xist* and resulted in an identification of multiple domains of secondary structure defined by variable and dynamic regions. Half of the *Xist* sequence forms well-defined structures, which could be targeted sites of signals from cellular environment (e.g., specific proteins) [59]. Xue et al. revealed the importance of lncRNA functional motifs in mechanisms of action, as they found out that deletion in 11 nt in an internal G-rich RNA motif (AGIL) of *Bvht* (*Braveheart*) impaired cardiomyocyte differentiation through a well-defined motif of secondary structure, regulating the cardiovascular lineage with cellular nucleic-acid-binding protein (CNBP) [60].

## 7. Involvement of Long Non-Coding RNAs at the Onset of Various Pathologies

After the regulation potential of lncRNAs was discovered, it was clear that these molecules are important in numerous cellular processes, including differentiation, cell death, protein metabolism, signal transduction, development processes, cell organization, RNA/DNA metabolism, stress response, cell adhesion, and intercellular signalization [6]. Thus, their deregulated expression is strongly associated with the development of several pathologies, especially neurodegenerative disorders, such as Alzheimer’s [61] and Huntington’s disease [62], cardiovascular diseases [63], and cancer [64]. Recently, it was shown that expression profiles of lncRNAs in cancer cells are significantly different from those in normal cells. In addition, some of them may be involved in metastatic processes. Generally, lncRNAs are strongly involved in all hallmarks of cancer, including proliferative signaling (*SRA*, *PCAT-1*, *KRASP1*), replicative immortality (*TERC*, *TERRA*), invasion and metastases (*MALAT1*, *HOTAIR*, *HULC*, *BC200*), angiogenesis (*ncR-uPAR*, *aHIF*), cell-death resistance (*PANDA*, *PINC*), and evasion of growth suppressors (*ANRIL*, *GAS5*, *LINC-p21*) [65]. Even though the mechanism of disease-causing events is not fully known yet, the function of lncRNAs as diagnostic, predictive, and prognostic biomarkers, as well as novel therapeutic targets in various pathologies, is undeniable [66].

## 8. Long Non-Coding RNAs in Multiple Myeloma

Even though the means of mechanisms for promoting tumor formation, progression, and metastasis are not fully elucidated [67], the abnormal expression profile of lncRNA in hematopoesis and blood diseases suggests their functional importance and potential in clinical applications. Long ncRNAs are involved in erythroid, myeloid, and lymphoid differentiation, and in the regulation of blood cell proliferation and survival, indicating their relevance in the pathogenesis of hematopoietic malignancies [68]. A large part of hematological neoplasias is represented by B-cell malignancies, which reflect specific stages of B-cell differentiation. There is accumulating evidence of lncRNA protagonists in normal human B-cell differentiation and in the pathogenesis of B-cell tumors. Moreover, 10% of all hematological malignancies are caused by the malignant proliferation of antibody-secreting PCs in BM [69].

Multiple myeloma is the second most common hematological malignancy characterized by pathological PC accumulation in BM with an incidence rate of 4.8 cases per 100,000 people in the Czech Republic [70], and 1.5 cases per 100,000 people worldwide [71]. MM is preceded by asymptomatic precancerosis MGUS with a risk of progression of 1% per year [72]. Events causing progression into MM are not clear, but genetic abnormalities, including mutations of tumor suppressors or oncogenes play an important role [73]. Multiple myeloma can later progress into extramedullary myeloma (EM), defined by extraskeletal infiltration of clonal PCs, or plasma cell leukemia (PCL), characterized by malignant cells leaving the BM microenvironment and circulating in peripheral blood [74]. Bone marrow microenvironment independence is a result of the accumulation of additional genetic mutations in malignant cells leading to aggressive stages of MM [75].

As remarkable progress in the treatment and diagnosis of MM was made and median survival doubled, a revision of the definition and staging was necessary. In 2014, the International Myeloma Working Group (IMWG) updated diagnostic criteria defined by the presence of any of the CRAB criteria, which describe organ damage (hypercalcemia (C), renal failure (R), anemia (A), and bone lesions (B)), by adding biomarkers to diagnose MM in patients without CRAB features. These biomarkers include the presence of clonal PCs in the BM in more than 60%, involved:uninvolved free light-chain ratio ≥ 100 mg/L, or >1 focal lesions on magnetic resonance imaging (MRI). These new criteria allowed for earlier diagnosis and initiation of therapy [1].

In 2016, a new staging system incorporated high-risk cytogenetic abnormalities to standard prognosis markers as the survival of MM patients depends on host factors, tumor burden, response to therapy, and tumor biology, including specific abnormalities, such as t(4;14), t(14;16), t(14;20), del(1p), del(17p), and gain(1q) influencing prognosis and disease course in MM. Identification of biomarkers of malignancy and developing a portfolio of clinical trials could provide the information necessary for early intervention and an improvement of median survival or the identification of a path to reliable MM treatment [76].

Multiple myeloma is a neoplasm of terminally differentiated B cells, which often have chromosomal abnormalities, such as translocations placing oncogenes under the control of immunoglobulin enhancers. This genomic instability leads to aberrant ploidy and structural rearrangements [77]. Half of MM cases are hyperploid, while the rest are associated with gene activation due to chromosomal translocations including the immunoglobulin heavy (*IGH*) locus at 14q32.33 [69]. It is known that many molecules and pathways are involved in MM pathogenesis, including non-coding RNA molecules [78]. The first studies concentrated on miRNA, and it was shown repeatedly that miRNAs are involved in MM pathogenesis [79,80,81]. In the past few years, the attention of researchers shifted to the role of lncRNAs in MM pathogenesis. Our group showed the deregulated expression of lncRNA *UCA1* in MM patients at diagnosis by quantitative polymerase chain reaction (qPCR) platform analysis. This lncRNA distinguishes MM patients from healthy donors with high sensitivity and specificity (85.0% and 94.7%, respectively). The *UCA1* levels correlated with albumin and monoclonal immunoglobulin serum levels, cytogenetic aberrations, and survival of MM patients [82]. Another lncRNA, *NEAT1*, was also found to be dysregulated with lower sensitivity and specificity. A recent study by Taiana et al. showed significant overexpression of *NEAT1* in MM patients in comparison to healthy donors by next-generation sequencing (NGS) followed by qPCR. Interestingly, it seems that dysregulation of *NEAT1* does not influence the prognosis of MM patients [83]. Another study showed high expression of *MALAT1* associated with progression from normal PCs into MM. Surprisingly, *MALAT1* expression did not correlate with clinical outcome of MM patients [37]. A different study using *GAPMER* technology showed that decreased expression of *MALAT1* decreased proliferation of MM cells. Moreover, decreased expression of *MALAT1* was connected to decreased expression of proteasome pathway genes. As proteasome inhibition was shown to be an important strategy for MM treatment [84], this study seems to suggest that targeting *MALAT1* might be a new treatment target for MM [85]. A unique study analyzed the expression of lincRNA and found an MM signature of these molecules that stratified MM patients. Together with protein-coding genes, lincRNA might be a useful tool for identification of ultra-high-risk patients [86]. A study by Cho et al. focused on lncRNA *MALAT1* in BM cells, suggesting that higher expression level of *MALAT1* could signify a connection with the BM microenvironment as a support system for MM cell proliferation. Expression levels of *MALAT1* changed during different stages of disease—patients with lower expression of *MALAT1* showed higher risk of early progression; thus, lncRNA *MALAT1* shows a potential of being a prognostic marker for these patients [58]. A study by Amodio et al. summarized the development of therapeutic agents targeting lncRNAs presented in tumors, including *MALAT1*, and showed significant advances. They referred to the establishment of *MALAT1*’s oncogenic role and its druggability by deletion of this lncRNA by zinc-finger nucleases, or by therapeutic targeting of *MALAT1* with synthetic oligonucleotides (siRNA, gapmeR) [87]. Abundant expression of *MALAT1* was detected in many human neoplasias, such as non-small-cell lung carcinoma, breast cancer, hepatocellular carcinoma, ovarian and cervical cancers, esophageal cancer, renal cell carcinoma, prostate cancer, osteosarcoma and Ewing sarcoma, multiple myeloma, mantle cell lymphoma, T-cell lymphoma, and many others. As it promotes proliferation and/or dissemination of tumor cells, the level of *MALAT1* correlates with tumor size, stage, and overall prognosis [87]. According to recent studies, lncRNA *MALAT1* is capable of upregulation of well-established epi-miRNA (subclass of tumor-suppressor miRNA with an ability to revert epigenetic aberrations) called miR-29b [88] characterized by inverse correlation with enhancer of zeste homolog 2 (EZH2) mRNA expression [89]. Inhibition of EZH2 led to upregulation of *miR-29b* as a result of reduced H3K27me3 in promotor regions of miR-29a/b-1 [89]. MiR-29b is naturally not the only overexpressed miRNA identified in MM. Calura et al. detected relevant miRNA/transcription factors of target gene regulation circuits linked to biological processes in MM. They identified a connection between specific transcription factors (*PBX1*, *CEBPA*) and a pathway-derived network of miR-99b/let-7e/miR-125a cluster upregulated in t(4;14) [90]. Zhou et al. determined that lncRNAs *RP4-803*, *RP1-43E13.2*, *ZFY-AS1*, and *RP11-553 L6.5* were capable of effective classification of patients into high-risk and low-risk groups depending on their expression correlating with overall survival (OS). These four lncRNAs are involved in initiation and progression of MM because of their association with genetic and epigenetic changes connected to MM. The role of lncRNA *MEG3* as a tumor suppressor was proposed as it is activated by tumor-suppressor protein p53. More than half of MM patients lost the expression of *MEG3*; it might play a role in osteogenic differentiation of mesenchymal stem cells (MSC) as it is disrupted in MM patients [36]. Shen et al. detected downregulation of *MEG3* linked with tumor progression of MM. A promising therapeutic target of *MEG3* was discussed in the context of *MEG3*/miR-181a/*HOAX* regulatory network. MiR-181a contends with MEG3, which acts as endogenous competitive RNA, to inhibit tumor progression. *MEG3*-associated sponging of miR-181a can lead to regulation of homeobox gene A11 (HOXA11) [91]. Protein-coding gene *ATG14* plays a crucial role in the autophagy pathway, which provides protection to cancer cells from stress initiated by radiation and chemotherapy. Another study showed that inhibition of miR-140-5p expression leads to contribution of *linc00515* to upregulation of *AGT14* in MM cells, which results in promotion of MM chemoresistance to melphalan, and vice versa; knockdown of *linc00515* leads to *AGT14* downregulation and inhibition of myeloma autophagy [92]. Long ncRNA *FEZF1-AS1*, associated with poor prognosis in MM patients, has a role of competing endogenous RNA in MM cells as it promotes proliferation via regulation of the miR-610/*Akt3* axis. *FEZF1-AS1* suppression arrested the cell cycle in the gap 0/1 (G0/G1) phase, inhibited proliferation and induced apoptosis; its higher level of expression, therefore, indicates MM progression [93]. A different study showed lncRNA *PRAL* in relation to overall survival (OS) in MM patients as multivariate Cox regression analysis determined that *PRAL* expression is an independent predictor factor for OS and disease-free survival (DFS). In this study, *PRAL* promoted cell-growth inhibition and apoptosis and increased bortezomib’s effect against MM via miR-210 targeting. It was also shown that, through miR-210 sponging, *PRAL* regulates the derepression of bone morphogenetic protein 2 (BMP2). This study revealed a key role of *PRAL*/miR-210/*BMP2* axis in MM pathogenesis and its association with the international staging system (ISS) stage and Durie–Salmon stage in MM patients [94]. Long ncRNA *CCAT1*, which was already reported in the development and progression of many malignancies, was the aim of another study that detected a correlation between the high expression level of *CCAT1* and shorter OS in MM patients. Positive regulation of HOXA1 expression via miR-181a-5p sponging showed an oncogenic role of *CCAT1* in MM as knockdown of *CCAT1* inhibited cell proliferation, promoted apoptosis, and suppressed tumor growth in vivo [95]. Apoptosis inhibition and cell-cycle progression were indicated in functional experiments in the study of Yang et al., who demonstrated high miR-410 expression in MM patients with shorter OS. Influence of this miRNA is mediated via direct downstream targeting of Krüppel-like factor 10 (KLF10), resulting in activation of the PTEN/PI3K/AKT pathway. MiR-410 is not only inversely correlated with KLF10, but also with lncRNA *OIP5-AS1* as accumulation of *miR-410*, induced by lncRNA *OIP5-AS1* loss, supported cell proliferation and other cellular behaviors mediated via the KLF10/PTEN/AKT axis in MM [96]. Another study showed high expression of lncRNA *CRNDE* in MM cells in relation to tumor progression and poor survival of patients. Further experiments using siRNA transfection indicated an increase in miR-451 expression and detected its negative correlation with this lncRNA. *CRNDE* negatively targets miR-451 by acting as a competing endogenous RNA [97]. Long ncRNA *H19*, a potential oncogene in many cancer types, was discussed in another study. The linkage between *H19* and the nuclear factor kappa B (NF-κB) pathway was suggested as a novel interpretation of the growth regulation mechanism of *H19* in MM. Short hairpin RNA (shRNA) transfection-induced *H19* knockdown inactivated the NF-κB pathway and inhibited the proliferation and viability of MM cells. Abnormally expressed *H19* in MM cell lines correlates with lower survival; treatment combining knockdown of this lncRNA and suppression of NF-κB provided synergistically inhibitory effects [98].

The MM cells use regulation molecules found in vesicles and exosomes for communication with other cells or tissues; lncRNAs are found in these vesicles as well [57]. Very few studies are published so far that concentrate on circulating lncRNAs. Isin et al. detected five differentially expressed lncRNA in the plasma of MM patients in comparison to healthy donors. Circulating lncRNA *HOTAIR*, *GAS5*, *MALAT1*, and *lincRNA-p21* showed lower level of expression, while lncRNA *TUG1* was upregulated. These results indicate that *TUG1* could play a role in the progression of MM [57]. Serum lncRNA *PCAT-1* showed significantly elevated expression in MM patients in comparison to healthy donors with high sensitivity and specificity (71.7% and 93.8%, respectively). A correlation with β2 microglobulin was also found [99]. Our own study showed dysregulation of circulating serum lncRNA *PRINS*. This lncRNA was able to distinguish MM and MGUS patients from healthy donors with a sensitivity of 84.9% and specificity of 83.3% [100]. An upregulated level of circulating serum lncRNA *H19* was found to be a biomarker of MM in early stages [101].

The overall description of expression and the functional role of specific lncRNAs, detectable in MM cells, are summarized in Table 1.

## 9. Conclusions

Multiple myeloma treatment underwent a major shift in the past 15 years as new drugs were included into standard treatment protocol leading to an unprecedented survival of MM patients. At the same time, new molecules involved in pathogenesis of this disease were identified, including non-coding RNA molecules. This review summarized current knowledge about these molecules. According to recent data, the evidence of lncRNA participating in the initiation and development of MM suggests clinical relevance in early diagnosis, prognosis, and possible therapeutic targeting of this disease. While many new exciting studies were published, more information that would clarify their role in the pathogenesis of this disease and possibly uncover new targets of therapy is needed.

## Figures and Tables

**Table 1:** Specific long non-coding RNAs found in multiple myeloma cells.

Specific lncRNA	Expression in MM vs. Normal	Results of Changed Expression in MM	Effect of Reversed Expression Profile	References
*UCA1*	↑	Cell cycle positive regulation via CREB regulation		[82]
*NEAT1*	↑	Increased DEX resistance	Sensitivity to DEX	[83]
*MALAT1*	↑	Increased proliferation	Decreased proliferation, proteasome inhibition	[37,58,84,85,86]
*MEG3*	↓	Negative effect to osteogenesis of mesenchymal stem cells (MSC)	MM–MSC differentiation	[36]
*linc00515*	↑	Chemoresistance	Inhibition of myeloma autophagy	[92]
*FEZF1-AS1*	↑	Increased cell proliferation	Cell inhibition, cell arrest, and apoptosis induction	[93]
*PRAL*	↓	Increased tumor growth	Tumor growth inhibition, promotion of apoptosis, and sensitivity to bortezomib	[87]
*CCAT1*	↑	Oncogenic role via positive regulation of HOXA1 expression	Cell proliferation inhibition, promotion of apoptosis, and tumor growth suppression in vivo	[94]
*OIP5-AS1*	↓	Increased cell proliferation via miR-410 accumulation		[96]
*CRNDE*	↑	Increased tumor progression via negative regulation of miR-451	Cell proliferation inhibition, promotion of apoptosis	[97]
*H19*	↑	Increased proliferation through NF-κB pathway	Inhibition of proliferation and viability	[98,101]
*TUG1*	↑	Promoting of proliferation, migration, and invasion	Suppression of cell proliferation, invasion, and colony formation	[57]

lncRNA: long non-coding RNA; MM: multiple myeloma; DEX: dexamethasone; CREB: cyclic AMP response element binding protein; MSC: mesenchymal stem cells; HOXA1: homeobox A1.
